# Evaluation of HPV16 E7 expression in head and neck carcinoma cell lines and clinical specimens

**DOI:** 10.1038/s41598-020-78345-8

**Published:** 2020-12-17

**Authors:** Koji Kitamura, Keisuke Nimura, Rie Ito, Kotaro Saga, Hidenori Inohara, Yasufumi Kaneda

**Affiliations:** 1 Division of Gene Therapy Science, Osaka University Graduate School of Medicine, Suita, Osaka 565-0871 Japan; 2 Department of Otorhinolaryngology-Head and Neck Surgery, Osaka University Graduate School of Medicine, Suita, Osaka 565-0871 Japan

**Keywords:** Biomarkers, Oncology

## Abstract

Human papillomavirus (HPV) 16 infection in the oropharynx is one of the major risk factors for oropharyngeal carcinoma. Although the HPV E6 and E7 proteins are known to have a role in head and neck carcinogenesis, whether their expression is maintained once the tumour has developed still remains unclear. We evaluated the expression of these proteins in HPV16-positive cancer cell lines and clinical oropharyngeal specimens. Two out of the four commercially available antibodies directed against the E7 protein could detect the E7 protein overexpressed in the 293FT cells, human embryonic kidney cells, although none of the four commercially available anti-E6 antibodies could detect the overexpressed E6 protein. Whereas HPV16-positive head and neck or cervical carcinoma cell lines expressed the E7 mRNA, the antibodies with an ability to detect the E7 protein could not detect it in western blotting in these HPV16-positive cell lines. In clinical specimens, E7 protein was partially detected in p16-positive area in p16-positive and HPV16 DNA-positive samples, but not in p16-negative and HPV DNA-negative or p16-positive and HPV DNA-negative samples. Consistent with these findings, the E7 protein was poorly translated from the endogenous structure of the E7 mRNA, although significant E7 mRNA expression was detected in these samples. Our findings indicate that E7 protein is partially expressed in p16-positive area in p16-positive and HPV16 DNA-positive clinical specimens.

## Introduction

Infection with high-risk human papillomavirus (HPV), especially HPV16, is a major risk factor associated with oropharyngeal squamous cell carcinoma (OPSCC) as well as cervical carcinoma^1^. In an earlier study, HPV prevalence worldwide is approximately 90% for cervical carcinoma and 30% for oropharyngeal carcinoma, particularly 72.2% for oropharyngeal carcinoma in developed countries^2–4^. While it has been well studied how HPV infection promotes cervical carcinogenesis, it is still unclear the mechanisms how HPV infection is related to oropharyngeal squamous cell carcinogenesis. Of approximately 40 HPV types that infect the anogenital mucosal epithelium, 12 types, namely HPV16, 18, 31, 33, 35, 39, 45, 51, 52, 56, 58, and 59, are most commonly found in carcinomas^5^. Thus, these HPVs have been classified as High-Risk HPVs (HR-HPVs). Notably, HPV16, which is an HR-HPV, is found in approximately 90% of the HPV-positive OPSCC cases^6^. The HPV E6 and E7 proteins are known to have a role in carcinogenesis. The HPV16 E6 and E7 genes, which are located closely at the beginning of the HPV genome, are initially transcribed as a single bicistronic pre-mRNA^7^. This bicistronic pre-mRNA includes three exons and two introns. Alternative RNA splicing generates some distinct mRNAs for E6 or E7 protein expression^8–11^. E6 and E7 act as oncoproteins, activating the cell cycle progression by promoting the degradation of p53 and the inactivation of retinoblastoma protein (pRb), respectively^12^. Therefore, targeting of E6 and E7 proteins has been investigated as a treatment option. Although a previous study has shown that the suppression of E6 or E7 mRNA in HPV16-related head and neck squamous cell carcinoma cell lines inhibits cell proliferation in vitro and tumour growth in animal models, it is still controversial whether sustained E6 or E7 expression is required in late stages of head and neck carcinogenesis^13,14^.

The immunohistochemical detection of p16 overexpression is clinically used as a surrogate biomarker of HPV infection in HPV-positive carcinomas^15^. However, the p16 immunohistochemistry (IHC) for HPV evaluation gives the discordance between p16 IHC and HPV DNA test results in some cases because of the absence of a direct mechanistic relationship between HPV DNA integration and p16 expression. Therefore, the aim of our research was to address whether E6 and E7 proteins can be detected in head and neck carcinoma cell lines and clinical specimens so that direct evaluation of their expression instead of indirect evaluation of p16 could be used to assess HPV infection in head and neck cancer specimens.

In this study, we detected E7-positive area in the IHC of clinical specimens, but the E7-positive area were found not to completely coincide with the p16-positive area. Our findings indicate that the E7 protein is partially expressed in p16-positive area in p16- and HPV16 DNA-positive clinical specimens. The results indicate the regulation of E7 protein expression in HPV-infected specimens.

## Results

### HPV16-positive cell lines express limited amounts of E7 protein that cannot be detected

**Figure 1:**
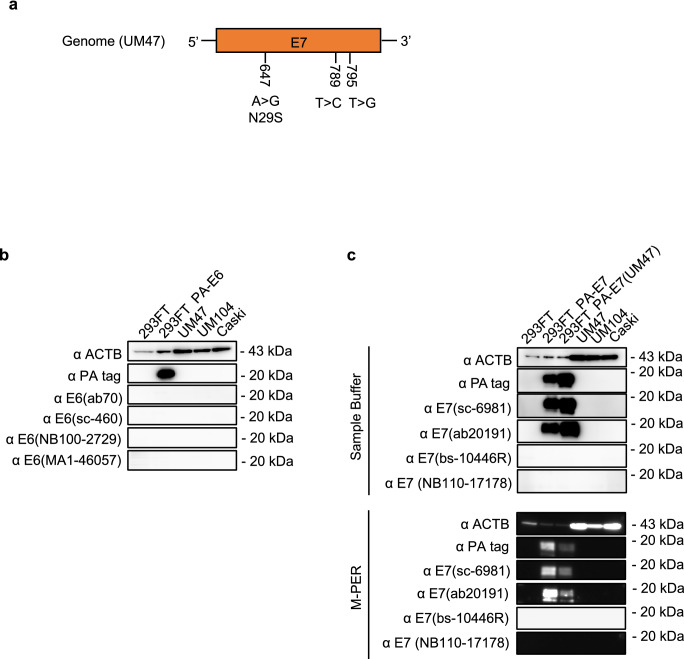
Detection of E6 and E7 proteins using specific antibodies against E6 and E7 in the head and neck carcinoma and cervical carcinoma cell lines. (**a**) The positions of the three mutations in the E7 sequence in UM47. (**b**,**c**) Western blotting of E6 (**b**) and E7 (**c**) proteins in 293FT cells (wild-type), 293FT cells transfected with pCAGIP-PA-E6/-PA-E7, and wild-type HPV16 ( +) cell lines (UM47, UM104, and Caski). (**c**) The E7 proteins were extracted using the sample buffer or with M-PER.

Because the functions of HPV E6 and E7 after carcinogenesis are not fully understood^16^, we first sought to evaluate the expression of E6 and E7 proteins in HPV16-positive cell lines (head and neck carcinoma cell lines, UM47 and UM104 and cervical carcinoma cell line, Caski). To express the E6 and E7 proteins as positive control, we used expression vector having plasmid constructs with the correct E6 or E7 sequences^17^ and the PA tag at their N-terminus (PA-E6, PA-E7). We also used the expression vector having plasmid construct with the UM47-E7 sequence and the PA tag at their N-terminus (PA-E7 (UM47)) because UM47-E7 sequence had three mutations in the E7 gene, whereas UM104 and Caski cell lines did not have any mutation (Fig. 1a).

To evaluate the expression of E6 and E7 protein, western blotting was performed using the extracts of HPV16-positive wild-type cells (UM47, UM104, Caski) and 293FT cells transfected with PA-E6, PA-E7, or PA-E7 (UM47) expression plasmids as positive controls. The E6 and E7 proteins were not detected in the HPV16-positive cell lines (Fig. 1b,c, Supplemental Fig. 1a,b). The anti-PA antibody was able to detect the PA-E6 protein in the 293FT cells transfected with pCAGIP-PA-E6, but the four anti-E6 antibodies could not detect the E6 proteins, including PA-E6 protein (Fig. 1b, Supplemental Fig. 1a). On the other hand, two of the four antibodies against the E7 protein could detect the PA-E7 protein in the 293FT cells transfected with pCAGIP-PA-E7 (Fig. 1c, Supplemental Fig. 1b). Immunofluorescence was also performed with UM104 and Caski using anti-E7 antibodies (sc-6981 and ab20191), but E7 protein was not detected (Supplemental Fig. 4a). These results indicated that the four anti-E6 antibodies could not detect the E6 protein and the two antibodies against the E7 proteins that could detect PA-E7 were not able to detect the E7 protein in the HPV16-positive cell lines and that the expression levels of the E7 protein in the HPV16-positive cell lines were not sufficient for detection by these antibodies.

### HPV16-positive cell lines express E6 and E7 mRNAs

**Figure 2:**
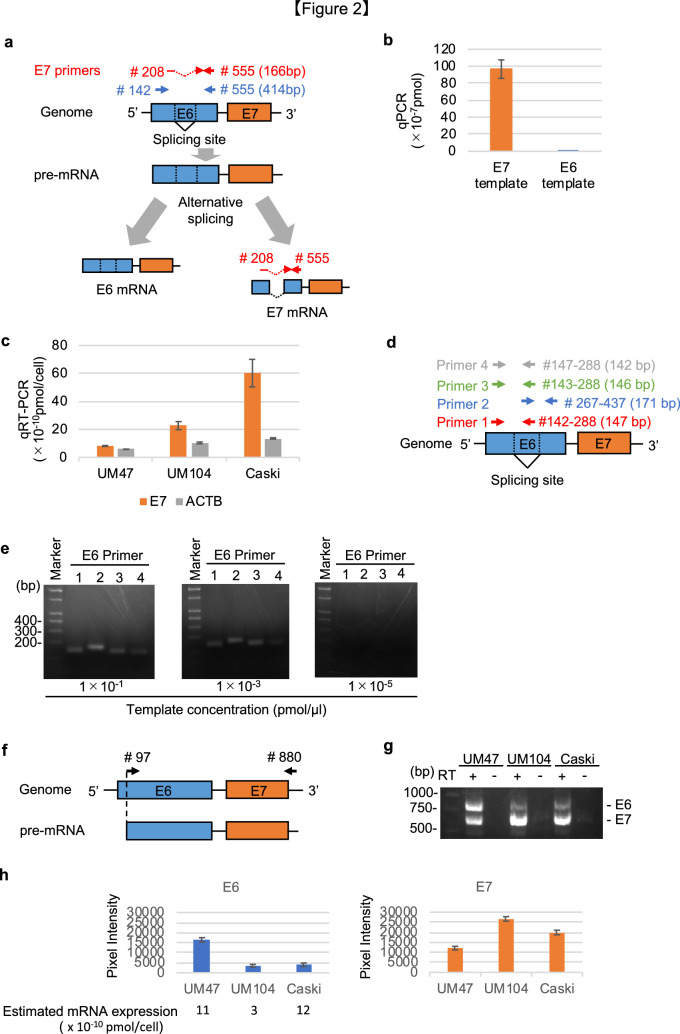
Evaluation of the expression of E6 and E7 mRNAs in head and neck carcinoma and cervical carcinoma cell lines. (**a**) The HPV16 E6 and E7 genes are initially transcribed as a single bicistronic pre-mRNA; the E6 or E7 mRNA is then generated by alternative splicing. The E7 primers were designed for binding within the E7 mRNA specific region near the splicing site (# 208 and # 555). (**b**) The specificity of E7 primer was confirmed using the pCR-Blunt E7 or E6 template. (**c**) The expression levels of E7 mRNA in the wild-type HPV16 (+) cell lines (UM47, UM104, and Caski) are shown. The data was normalized to cell numbers. (**d**) Four E6 primer sets were designed to bind within the E6 mRNA specific region. (**e**) The results of PCR using the four E6 primer sets and three different concentrations of the E6 template. (**f**–**h**) The cDNA from the UM47, UM104, and Caski cells were amplified using # 97 and # 880 primers recognizing the pre-mRNA. RT+, reactions performed in the presence of reverse transcriptase. RT−, Reactions performed in the absence of reverse transcriptase. The bars represent the intensities of the amplified E6 and E7 bands.

The HPV16 E6 and E7 genes are located close to each other at the beginning of the HPV genome^17^. These two genes are transcribed as a single bicistronic pre-mRNA, which is processed into some distinct mRNAs for E6 or E7 protein expression through alternative RNA splicing^8–11^ (Fig. 2a). To evaluate the E6 and E7 mRNA expression in the HPV16-positive cell lines, we designed specific primers for the E6 and E7 genes (Fig. 2a # 142 and # 555; # 208 and # 555). The E6 and E7 polymerase chain reaction (PCR) products amplified using these primers were introduced into plasmids to generate control templates. The E7 primers amplified the E7, but not the E6, template with the concentration of 1 × 10^–5^ pmol/µL (Fig. 2b). The absolute expression level of the E7 mRNA in each cell was determined in qRT-PCR using the E7 primer, resulting in 8 × 10^–10^ pmol/cell for UM47, 23 × 10^–10^ pmol/cell for UM104 and 60 × 10^–10^ pmol/cell for Caski (Fig. 2a # 208 and # 555 and Fig. 2c). It indicated that the expression level of E7 mRNA was comparable to that of ACTB, although the E7 protein was not detected in the HPV16-positive cell lines. These results provide an evidence for the expression of E7 mRNA in the HPV16-positive cell lines.

Next, to specifically detect the E6 mRNA, we designed four E6 primer pairs; one of these primers was specifically designed against a sequence within the splicing site (Fig. 2d). Although these E6 primers amplified E6 at more than 1 × 10^–3^ pmol/µL concentration of the E6 template, they could not do so at a template concentration of 1 × 10^–5^ pmol/µL (Fig. 2e, Supplemental Fig. 2a). The template with the concentration of 1 × 10^–5^ pmol/µL was enough to detect E7 and ACTB expression by qRT-PCR (Fig. 2c), suggesting that it was difficult to evaluate the E6 mRNA expression in the HPV16-positive cell lines at the same concentration of E7 template by qRT-PCR at least using these four E6 primers.

We, therefore, performed qRT-PCR using primers from both the ends of the pre-mRNA (Fig. 2f, # 97 and # 880) for UM47, UM104, and Caski and electrophoresed the PCR products on an agarose gel (Fig. 2g, Supplemental Fig. 2b). The amplified bands were quantitatively analysed by densitometry to evaluate the E6 and E7 mRNA expression levels (Fig. 2h); the densitometric analysis was done taking care that the intensity of bands was not saturated. We observed that the E6 and E7 mRNAs were expressed in the UM47, UM104, and Caski cells. The expression levels of E6 were estimated to be 11 × 10^–10^, 3 × 10^–10^, and 12 × 10^–10^ pmol/cell in the UM47, UM104, and Caski cells, respectively (Fig. 2h). These data indicated that E6 and E7 mRNA are sufficiently expressed in each cell line compared with the housekeeping gene ACTB.

### E7 protein is found partially in the p16-positive area in p16-positive and HPV16 DNA-positive clinical specimens

**Figure 3:**
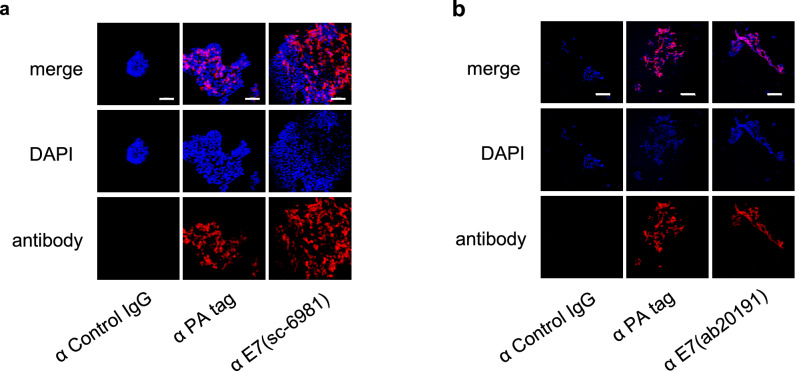
Immunofluorescence analysis of E7 protein using 293FT cells with the overexpressed E7 proteins. (**a**,**b**) Anti-E7 antibodies (**a** sc-6981 and **b **ab20191) that were able to detect the E7 protein in western blotting were used for immunofluorescence analysis of 293FT cells transfected with pCAGIP-PA-E7. DAPI was used for nuclear staining. Scale bar 100 μm.

Although HPV E6 and E7 proteins are known to have a role in carcinogenesis^13,14^, it remains controversial whether these proteins are still expressed once the carcinoma has developed. To determine the levels of E7 proteins in HPV16 DNA-positive carcinoma cells, we first tested whether the two E7-specific antibodies that could detect the E7 protein in western blotting could detect the overexpressed E7 proteins in 293FT cells by immunofluorescence staining (Fig. 3a,b). The two anti-E7 antibodies could detect the PA-E7 protein by immunofluorescence staining.

**Figure 4:**
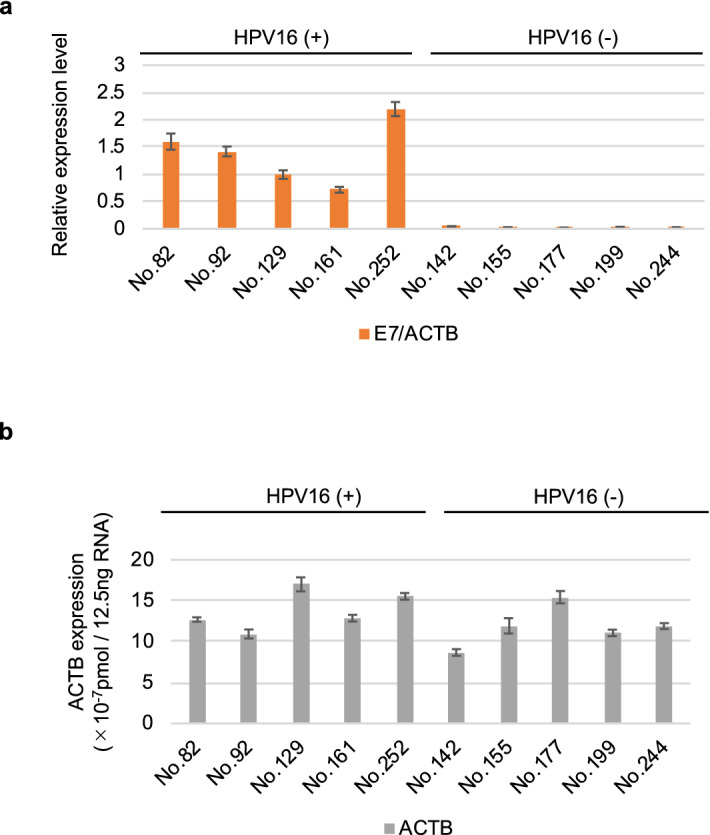
qRT-PCR analysis of mRNA expression in clinical samples of HPV16 (+) and HPV16 (−) oropharyngeal carcinoma. (**a**) Relative expression of E7 in clinical samples s of HPV16 (+) or HPV16 (−) oropharyngeal carcinoma. The values were normalized to that obtained for ACTB. (**b**) ACTB expression in the samples was determined to be 10^–7^ pmol/12.5 ng RNA.

The evaluation of the HPV16 DNA-positive or HPV-negative status of clinical specimens was done by PCR amplification of L1, E6, and E7 using differences in their sequence (data not shown). To confirm whether the E7 mRNA was actually expressed in the HPV16 DNA -positive clinical specimens and was not expressed in the HPV-negative specimens, we performed qRT-PCR using the appropriate primers (Fig. 2a, #208 and #555). We observed that the expression of E7 mRNA was comparable to that of ACTB mRNA in these clinical specimens (Fig. 4a,b).

**Figure 5:**
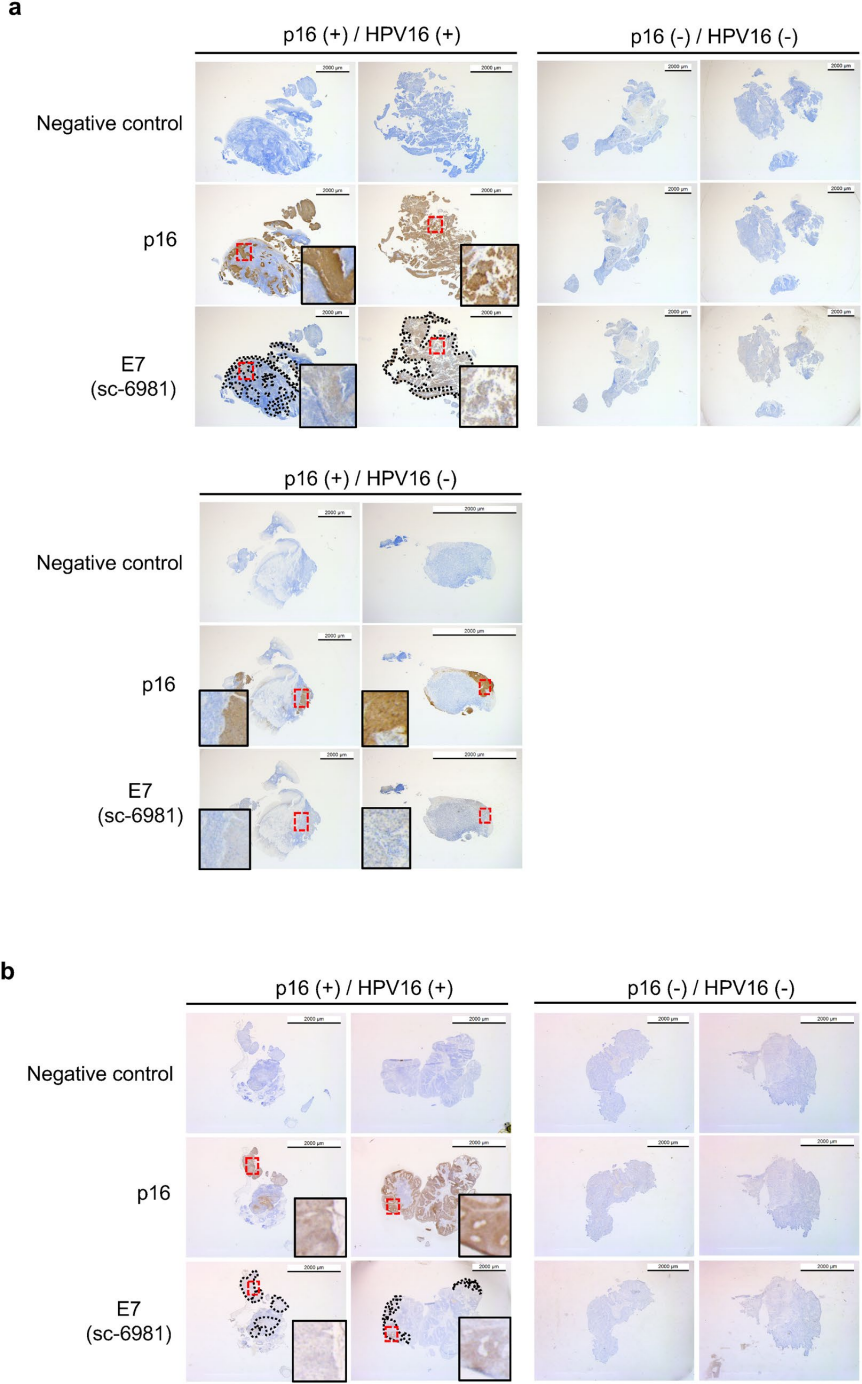
Immunohistochemical analysis of E7 protein in clinical samples of HPV16 (+) and HPV16 (−) oropharyngeal carcinoma. (**a**) Formalin-fixed and paraffin-embedded samples were stained using the indicated antibodies. (**b**) Frozen samples were stained using the indicated antibodies. Scale bar 2 mm.

We next examined the localization of the E7 protein and its expression levels in clinical specimens by immunohistochemical staining. In formalin-fixed and paraffin-embedded (FFPE) sections, the protein was detected in a part of the p16-positive area (surrounded by a dotted line in the figure) of p16-positive and HPV16 DNA -positive clinical specimens, but not in p16-positive and HPV-negative or in p16-negative and HPV-negative clinical specimens (Fig. 5a). We also performed immunohistochemical staining using samples without formalin fixation and paraffin embedding, because some antibodies do not work well with FFPE sections. Indeed, E7 was also detected in a part of p16-positive area in the frozen sections of fresh clinical specimens after paraformaldehyde fixation, with the same pattern as observed in FFPE sections (Fig. 5b). IHC was also performed with the anti-E7 antibody (sc-6981) in cervical carcinoma and normal cervical tissue, and the results were similar to those for oropharyngeal carcinoma (Supplemental Fig. 5a). These data showed the ability of the anti-E7 antibody to detect the E7 protein in clinical specimens of oropharyngeal carcinoma and indicated that E7 protein is found partially in the p16-positive area in p16-positive and HPV16 DNA-positive samples.

### E7 protein is poorly translated from the endogenous structure of E7 mRNA

**Figure 6:**
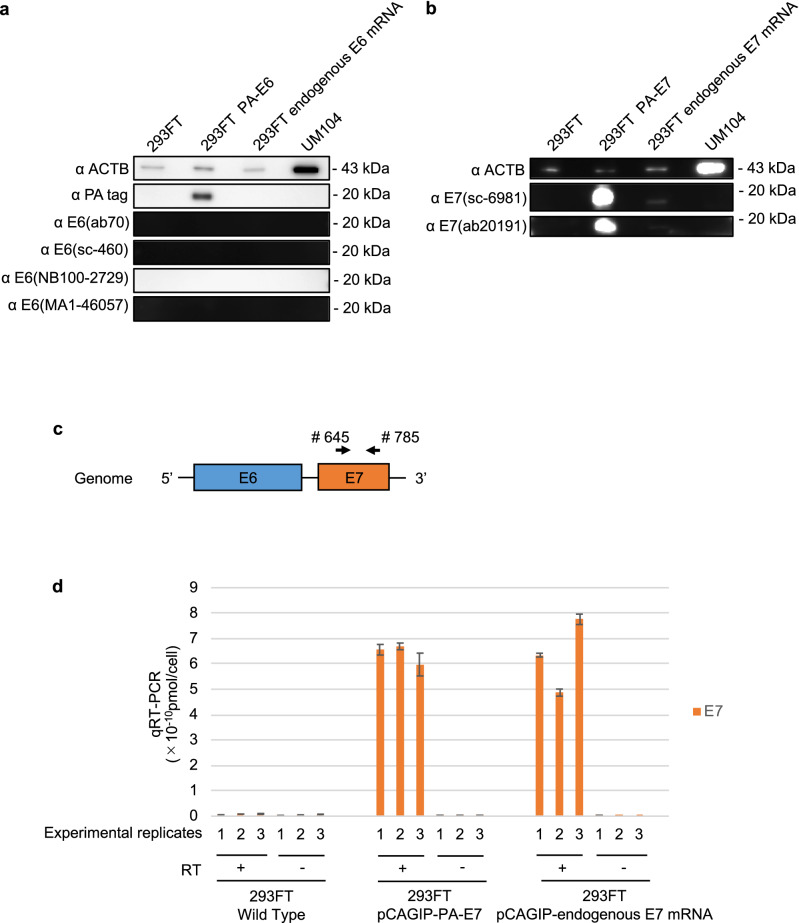
E7 protein expression in 293FT cells by overexpression of E7 mRNA having the endogenous structure. (**a**,**b**) Western blotting of E6 (**a**) and E7 protein (**b**) in 293FT cells (wild-type), 293FT cells transfected with pCAGIP-PA-E6/-PA-E7 or pCAGIP-endogenous E6/E7 mRNA, and UM104 cells. (**c**) E7 primers (# 645 and # 785) designed for binding within the E7 gene for use in qRT-PCR. (**d**) qRT-PCR analysis of E7 expression in 293 FT cells transfected with pCAGIP-PA-E7 or -endogenous E7 mRNA plasmids. The values for E7 expression per cell are shown. RT+, Reactions performed in the presence of reverse transcriptase. RT−, reactions performed in the absence of reverse transcriptase. The numbers on the x-axis show the experimental replicates.

The results of immunohistochemical staining indicated that the expression of the E7 protein was limited in comparison to that of p16, although substantial expression of the E7 mRNA was detected in these clinical specimens using qRT-PCR. These data indicate the possibility of translational control of the E7 mRNA also in HPV16-positive cells.　Therefore, we examined whether the high endogenous E7 mRNA expression leads to the high E7 protein translation. Western blotting was performed using 293FT cells transfected with expression vector having the full-length E6 or E7 cDNAs amplified using PCR (Fig. 2f, # 97 and # 880), meaning the expression vector have the full-length E6 or E7 mRNA sequence. The results of western blotting revealed that the endogenous structure of E7 mRNA produced little amounts of the E7 protein, compared to the protein produced by the E7 mRNA containing the Kozak sequence (PA-E7), whereas the E6 protein was not detected for both the endogenous structure and the Kozak sequence containing PA-E6 mRNA (Fig. 6a,b, Supplemental Fig. 3a,b). We also evaluated the E7 mRNA expression level in the transfected 293FT cells using qRT-PCR with primers designed to bind within the E7 gene (Fig. 6c). The expression of the E7 mRNA was comparable in all the transfected 293FT cells (Fig. 6d). These data indicated that the E7 protein was poorly translated from the endogenous structure of E7 mRNA in 293FT cells.

## Discussion

The HPV infection in the oropharynx is one of the risk factors for oropharyngeal carcinoma. Many studies have reported the association of E6 and E7 oncoproteins with carcinogenesis^18^. However, it remains unclear whether these proteins are involved in the maintenance of oropharyngeal carcinoma after carcinoma has formed, because there are scant evidences for the expression of these proteins in oropharyngeal carcinoma. The present study revealed that the E6 protein was not detectable with any of the tested anti-E6 antibodies and that the E7 protein was partially detected in p16-positive and HPV16 DNA-positive clinical specimens, although significant E7 mRNA expression was detected in these samples. The PA-E6 protein was highly translated in 293 FT transfected with PA-E6, but the tested anti-E6 antibodies failed to detect the E6 protein (Fig. 1b). These results suggest that the tested anti-E6 antibodies have no ability to detect E6 protein by western blotting. Since E6 protein was not detected by western blotting, IHC for E6 protein was not performed. Thus, it remains unclear whether the E6 protein is expressed in clinical specimens and cell lines. As for the E7 protein, a previous study showed E7 protein expression in UM47 by western blotting using M-PER as a lysis buffer^19^. We performed western blotting with M-PER in the same conditions as with sample buffer, but E7 protein could not be detected in both condition (Fig. 1c). Our results imply a mechanism that regulates the translation efficiency from viral mRNA. As a result supporting the hypothesis, our data suggest that the limited E7 protein expression might be due to the poor translation efficiency of the endogenous E7 mRNA in cultured cells. There are some limitations in the present study. Although several spliced forms of E6 or E7 mRNA are known^20^, the limited form was detected in the present study because the concentration might be too low for other forms to be detected by PCR (Fig. 2g). Because we were unable to get enough amounts of clinical samples, we could not perform in situ hybridization (ISH) to detect the localization of the E7 mRNA in HPV16 DNA-positive clinical specimens. Furthermore, western blotting was difficult to perform using the HPV-positive samples. Results from cell lines and clinical specimens cannot be evaluated equally. Careful attentions may be required for the interpretation of past studies that use HPV anti-E6 or anti-E7 antibodies because our results suggest a possibility of the limited expression of the HPV proteins in the HPV-positive cancer cell lines.

Several studies have demonstrated improved survival rates in HPV-positive OPSCC patients compared with those in HPV-negative patients^21–25^. Consequently, less intensive treatment protocols are expected to achieve good outcomes, with less toxicity. Several de-escalation treatments have recently been conducted in clinical trials^26^. Thus, we need a reliable and practical testing strategy to identify the truly HPV-positive OPSCC and to eliminate the discordance between p16 IHC and HPV DNA test results.

Currently, p16 IHC is clinically used as a surrogate biomarker of HPV-positive carcinomas in biopsies^27–29^. However, because the p16 IHC is not a direct method for detecting the HPV-derived proteins, there are some cases that have the discordances between p16 IHC and HPV DNA test results, such as p16-positive and HPV DNA-negative. Thus, a direct method to detect the HPV-derived proteins in clinical specimens is desirable to evaluate the HPV infection. The HPV oncoproteins, E6 and E7, are known to be responsible for carcinogenesis^12^. However, the presence of these proteins in carcinomas remains unclear. In the present study, we demonstrated that the E6 protein could not be detected by any of the tested anti-E6 antibodies (Fig. 1b) and low amounts of the E7 protein were found partially in p16-positive area in p16-positive and HPV16 DNA-positive samples, although the E7 mRNA was substantially expressed (Figs. 4a, 5a,b). PCR and ISH can be used to detect the HPV16. Although PCR is a sensitive method for detecting HPV^16^, it cannot distinguish the mere presence of HPV from HPV infection that results in carcinogenesis. ISH is not a very sensitive method to detect the HPV RNA^16^. Furthermore, ISH detects the RNA but not the protein. Thus, p16 IHC might be the most valuable method to identify HPV-positive cases.

The role of E7 in epithelial cells in the process of carcinogenesis has been investigated in previous studies in cervical carcinoma^14,30^. The E7 protein plays a pivotal role in creating a conducive environment for replication of the viral genome, because the HPV genome does not code for the enzymes required for viral replication. Thus, HPV utilizes the replication system of host cells to amplify its genome^31^. The E7 protein is known to work cooperatively with the E6 protein, resulting in the transformation of epithelial cells to carcinoma cells. However, the role of E7 protein in tumour maintenance has not been fully elucidated for oropharyngeal carcinoma.

The E7 protein expression in clinical specimens has been evaluated in few studies. A previous paper, although it analyzed only one OPC specimen, still observed E7 expression by IHC^32^. Our results revealed that the HPV16-positive cell lines expressed the E7 mRNA significantly, but the E7 protein was not detected in western blotting and immunofluorescence staining and that the HPV16-positive clinical specimens also expressed the E7 mRNA significantly, but the E7 protein was detected only partially within the p16-positive area (Figs. 1c, 2c, 4a, 5a,b and Supplemental Fig. 4a).

The E7 protein transforms the normal epithelial cells to carcinoma cells^14^. Some previous studies have shown that the knockdown of E7 fails to initiate cellular proliferation^33^ and that the HPV genome lacking E7 fails to induce DNA synthesis or markers of active cell cycle in suprabasal epithelial layers^34,35^. The E7 protein disrupts the function of pRb leading to transcriptional activation of a number of cell cycle-related genes by the released E2F, thus, stimulating the G_1_ to S phase transition^36,37^. Recent studies have revealed that in addition to the pRb/E2F system, the E7 protein also affects other transcription factors, such as NF-κB, and IFN pathways, TGF-β signalling, and histone deacetylases (HDACs)^30,38–46^. This suggests that various factors cause significant transcriptional changes in HPV-infected cells and lead to immortalization and genome instability, and eventually to carcinoma. Considering the results reported previously and based on our own results of poor expression of the E7 protein, HPV-infected carcinoma cells are expected to depend on various other factors for maintaining the tumour.

Viral mRNAs, including the HPV mRNA, are translated using the host’s cellular translation system. In the translation of influenza virus mRNAs, it is known that the viral RNA polymerase removes the cap structure from the mRNAs of host cells and binds to viral mRNAs for use as a primer. Transcription and translation are initiated, using the host-cell translation mechanisms^47^. In the case of HCV, translation is initiated, using the internal ribosome entry site in the 5′-region of mRNAs^48^. However, the mechanism from transcription to translation in HPV is not yet clear.

In the present study, we demonstrate that the E7 protein was poorly translated from the overexpressed E7 mRNA with endogenous structure in the 293FT cells, although the E7 mRNA with the endogenous structure was comparably expressed as did the E7 mRNA containing the Kozak sequence (Fig. 6). These results suggest that there might be a regulator at the translation initiation stage, because the E7 protein was sufficiently translated from the overexpressed pCAGIP-PA-E7 in the 293FT cells. Indeed, HPV has a mechanism for regulating the E6 and E7 transcription levels through the viral repressor, E2^49^. The mechanism prevents the production of E6 and E7 proteins in the infected squamous basal cells. After escaping the E2 repressor function and differentiating the cells into the upper epithelial layers^50–53^, the HPV-infected cells start the production of E6 and E7 proteins. Thus, there might be some regulators that control the translation of HPV-positive mRNAs in the epithelial cell-specific or HPV genome-encoding factors. However, another possibility is that the E7 protein may be unstable and subject to degradation. It should also be noted that there are histological differences between the reticulated epithelium of the tonsillar crypts, the origin of oropharyngeal carcinoma, and the cervical epithelium, the origin of cervical carcinoma. It is necessary to consider that the differences in these tissues affect the expression and regulation of E6 or E7 proteins for carcinogenesis and subsequent development. These suggest that the further elucidation of the E7 protein functions once the tumor has developed may be required to target E7 protein for therapy.

In summary, we investigated the E7 expression at mRNA and protein level in HPV16-positive cell lines and clinical specimens. Our results demonstrate that the HPV16-positive cell lines significantly express the E7 mRNA, but not the E7 protein, and that HPV16-positive clinical specimens also significantly expressed the E7 mRNA, but expressed the E7 protein partially within the p16-positive area. Consistent with these findings, the E7 protein was poorly translated from the endogenous structure of the E7 mRNA. Our findings suggest the existence of a mechanism that regulates the efficiency of translation from the viral mRNA.

## Methods

### Ethical approval and informed consent

We obtained the informed consent of patients to analyse the clinical specimens. The study protocol was approved by the Office of Research Ethics of Osaka University (approval number: 12391 and 10064). All the methods were performed in accordance with the relevant guidelines and regulations.

### Cell lines and cell culture

UM47 and UM104, the HPV16-positive head and neck carcinoma cell lines, were purchased from The University of Michigan. Caski was the gift from Department of Obstetrics and Gynecology, Osaka University Graduate School of Medicine. UM47 and UM104 were derived from lateral tongue carcinoma and oral cavity carcinoma respectively. These cell lines were cultured in Dulbecco’s modified Eagle’s medium (DMEM, Nacalai Tesque), supplemented with 10% foetal bovine serum (FBS, Sigma), 1% non-essential amino acids (NEAA, Nacalai Tesque), 1% L-glutamine (Nacalai Tesque), and 1% penicillin/streptomycin solution (Nacalai Tesque). The HPV16-positive cervical carcinoma cell line, Caski, was cultured in DMEM, supplemented with 10% FBS and 1% penicillin/streptomycin. The human embryonic kidney cells, 293FT, were cultured in DMEM, supplemented with 10% FBS, 1% NEAA, 1% L-glutamine, and 1% sodium pyruvate (Sigma). These cells were incubated at 37 °C in a humidified atmosphere of 95% air and 5% CO_2_.

### Transfection

The 293FT cells were transfected with pCAGIP-PA-E6 or pCAGIP-PA-E7 for overexpression of E6 or E7, or with pCAGIP-E6 or pCAGIP-E7 encoding the endogenous structure of E6 and E7 for the overexpression of endogenous E6 or E7 mRNA. Fugene HD (Promega) was used to transfect the cells. The 293FT cells were seeded into six-well plates at a density of 2 × 10E5 cells per well, to which 3 µg/well DNA plasmid in Opti-MEM (Gibco) and 9 µL/well Fugene HD were added. The transfected cells were incubated at 37 °C in a humidified atmosphere of 95% air and 5% CO_2_ for 48 h.

### Plasmid construction

The E6 and E7 cDNA were amplified from UM47 and UM104 cells with 5′-CACCATGCACCAAAAGAGAACTGC-3′ (# 83) and 5′-TTACAGCTGGGTTTCTCTAC-3′ (# 559) primers for E6 and with 5′-CACCATGCATGGAGATACACCTAC-3′ (# 562) and 5′-TTATGGTTTCTGAGAACAGA-3′ (# 858) primers for E7. The cDNA was generated by SuperScript III (Thermo Fisher). The endogenous structures of E6 and E7 mRNA were amplified from UM104 with 5′-CACCAACTGCAATGTTTCAGGACCCA-3′ (Fig. 2f, # 97) and 5′-CTGCAGGATCAGCCATGGTAGA-3′ primers (Fig. 2f, # 880). E6 or E7 cDNAs were introduced into pCAGIP-PA-gw and the endogenous E6 and E7 mRNA into pCAGIP-gw, using the Gateway technology (Thermo Fisher). The nucleotide positions of the primers were determined with reference to Papillomavirus Episteme^17^.

### Western blotting

The proteins were extracted from 1 × 10E5 cells in 10 µL sample buffer or 5 µL M-PER (Thermo Scientific). The extracted proteins were loaded on 10–20% polyacrylamide gradient gel (Wako) and transferred onto a polyvinylidene difluoride membrane. The membrane was blocked with 3% skimmed milk at room temperature for 1 h, and then incubated with one of the anti-HPV16 E6 (ab70 #GR247586-15, #GR3225702-2, Abcam, 1:100 dilution; sc-460 #D0115, Santa Cruz, 1:100 dilution; NB100-2729 #A-5, Novus, 1:100 dilution; MA1-46057 #SI2446982A, Invitrogen, 1:100 dilution), anti-HPV16 E7 (ab20191 #GR120242-15, #GR120242-20, Abcam, 1:100 dilution; sc-6981 #B0215, Santa Cruz, 1:100 dilution; NB110-17178 #16/03-HP16-281, Novus, 1:100 dilution; bs-10446R #AC12312777, Bioss, 1:100 dilution), or anti-beta actin (A5441, Sigma, 1:5000 dilution) antibodies. After washing the membrane two times and blocking with 3% skimmed milk, it was incubated with HRP-conjugated mouse secondary antibody. The signals were detected with Chemi-Lumi One or Chemi-Lumi One Super (Nacalai Tesque) using an ImageQuant LAS 4000 mini system (GE Healthcare).

### Quantitative RT-PCR

Total RNA was extracted from confluent cells or surgically removed clinical fresh specimens using ISOGEN (Nippon Gene) following homogenization with Multi-Beads Shocker (Yasui Kikai). 500 ng of RNA was used for the preparation of cDNA and reverse transcription was performed with SuperScript ƖƖƖ (Invitrogen). The PCR was performed using SYBR Green in a C1000 Thermal Cycler (Bio-Rad). We used the appropriate primers (Fig. 2a, #208 and #555) in Figs. 2b,c and 4a, and primers (Fig. 6c, # 645 and # 785) in Fig. 6d, respectively. To make the specific primer for E7 gene (Fig. 2a, #208 and #555), one of the E7 primers was designed to straddle over the splicing site in the E7 mRNA to specifically detect it, because the difference between the E6 and E7 mRNAs is in the splicing (Fig. 2a). The starting quantity was 0.84 × 10^–5^ pmol for E7 and 1.87 × 10^–5^ pmol for ACTB to prepare the standard curve. The values were normalized to cell numbers or ACTB. The absolute quantification of RNA values per cell was done, using the PCR products whose concentrations were measured as the standard.

### Immunofluorescence staining

The 293FT, UM104 and Caski cells (2 × 10E5) were seeded in 35 mm glass-bottom dishes (MatTek Corporation). After 24 h, these cells were transfected with pCAGIP-PA-E6 or pCAGIP-PA-E7 using Fugene HD and cultured for 48 h. After washing with PBS, these cells were fixed with 4% paraformaldehyde (PFA) for 5 min at room temperature. After permeabilisation of these cells with 0.5% Triton-X 100 for 10 min at room temperature, these cells were blocked with 5% skimmed milk for 30 min at room temperature, and then incubated with the primary antibodies: anti-E7 (ab20191, Abcam, 1:100 dilution; sc-6981, Santa Cruz, 1:50 dilution), anti-PA tag (NZ-1, Wako, 1:100 dilution), or anti-mouse control IgG (ab18413, Abcam, 1:100 dilution) at 4 °C overnight. The goat anti-mouse IgG conjugated with Alexa Fluor 546 (Invitrogen) was used as a secondary antibody for E7 and the mouse control IgG or goat anti-rat IgG conjugated with Alexa Fluor 546 (Invitrogen) were used as secondary antibodies for the PA tag. The pictures of the immunofluorescently stained cells were captured with a confocal laser microscope (A1Rsi/Ti-PF, Nikon).

### Immunohistochemical analysis

Cervical cancer and normal cervical tissue were obtained from Department of Obstetrics and Gynecology, Osaka University Graduate School of Medicine.

For oropharyngeal cancer, we analyzed two FFPE clinical specimens in p16-positive and HPV16 DNA-positive samples, in p16-positive and HPV16 DNA-negative samples and in p16-negative and HPV16 DNA-negative samples, and two frozen clinical specimens in p16-positive and HPV16 DNA-positive samples and in p16-negative and HPV16 DNA-negative samples. We also analyzed three FFPE clinical specimens in cervical cancer and normal cervical tissue. The FFPE blocks of surgically resected oropharyngeal carcinoma specimens were sectioned to a thickness of 2–3 µm. The sections were de-paraffinised and rehydrated in xylene and a graded-series of alcohol. Antigen retrieval in the FFPE sections was carried out using Histofine (code 415201, Nichirei Biosciences Inc.) by microwave treatment at 95 °C for 20 min.

The biopsy samples of oropharyngeal carcinoma were embedded in Tissue-Tek O.T.C. compound (Funakoshi) at − 80 °C for obtaining fresh frozen sections. These frozen tumours were sectioned to a thickness of 6 µm. The sections were dried at room temperature for 10 min and fixed for 15 min using 4% PFA; antigen retrieval was not done for these sections.

For both the types of sections, after washing with PBS for 5 min, endogenous peroxidase was inactivated with hydrogen peroxide [Dako EnVision + System-HRP kit (Dako) for E7 and CINtec Histology Kit (Roche) for p16] for 5 min and the non-specific proteins were blocked using Protein Block (X0909, Dako) for 30 min. After washing with PBS for 5 min, both the types of tumour sections were covered with anti-E7 (sc-6981, Santa Cruz, 1:50 dilution) or anti-p16 antibody or with a negative control reagent (CINtec Histology Kit, Roche, undiluted solution) for 60 min. After three washes with PBS, the tissue sections were covered with the peroxidase labelled polymer for 30 min. After three washes with PBS, the DAB substrate chromogen solution was added onto the sections and incubated for 1 min. Subsequently, the sections were washed under running water for 5 min, and counterstaining was done for 30 s with haematoxylin (S3301, Dako). After washing under running water for 5 min, the FFPE sections were dehydrated in a graded series of alcohol and xylene.

### Densitometric analysis

The images obtained after electrophoresis of the PCR products were analysed using the Image J software.

## Supplementary Information

Supplementary Information

## Data Availability

The datasets generated and/or analysed in this study are available from the corresponding author on reasonable request.
