# Author Correction: Evaluation of HPV16 E7 expression in head and neck carcinoma cell lines and clinical specimens

**DOI:** 10.1038/s41598-021-91694-2

**Published:** 2021-06-04

**Authors:** Koji Kitamura, Keisuke Nimura, Rie Ito, Kotaro Saga, Hidenori Inohara, Yasufumi Kaneda

**Affiliations:** 1 Division of Gene Therapy Science, Osaka University Graduate School of Medicine, Suita, Osaka 565-0871 Japan; 2 Department of Otorhinolaryngology-Head and Neck Surgery, Osaka University Graduate School of Medicine, Suita, Osaka 565-0871 Japan

Correction to: *Scientific Reports* https://doi.org/10.1038/s41598-020-78345-8, published online 17 December 2020

This Article contains an error in Figure 1 where in M-PER, western blotting results other than α E7 (bs-10446R) are inverted black and white in panel (C). The correct Figure 1 appears below as Figure 1.

**Figure 1:**
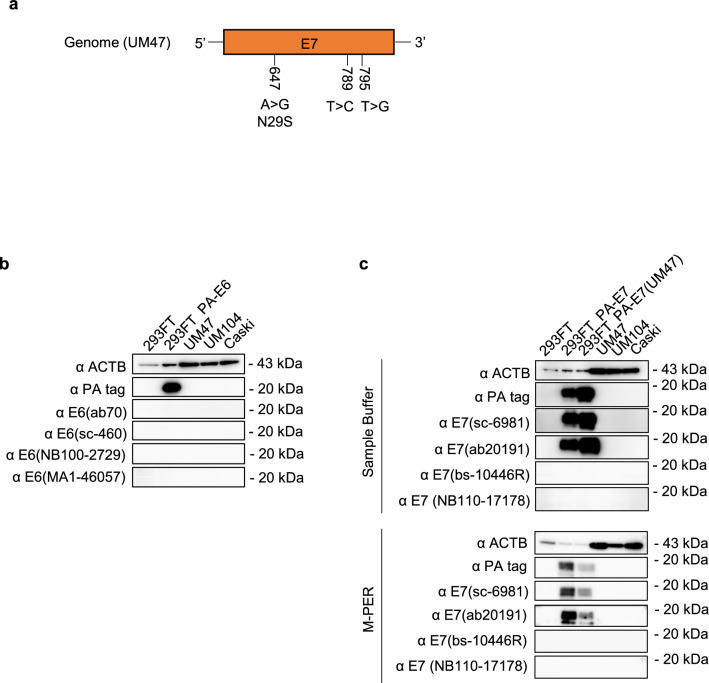
Detection of E6 and E7 proteins using specific antibodies against E6 and E7 in the head and neck carcinoma and cervical carcinoma cell lines. (**a**) The positions of the three mutations in the E7 sequence in UM47. (**b**,**c**) Western blotting of E6 (**b**) and E7 (**c**) proteins in 293FT cells (wild-type), 293FT cells transfected with pCAGIP-PA-E6/-PA-E7, and wild-type HPV16 (+) cell lines (UM47, UM104, and Caski). (**c**) The E7 proteins were extracted using the sample buffer or with M-PER.

Secondly, Figure 6 contains an error, where the western blotting results for α E6 (ab70), α E6 (sc-460) and α E6 (MA1-46,057) are inverted black and white in panel (A) and all western blotting results are inverted black and white in panel (B). The correct Figure 6 appears below as Figure 2.

**Figure 2:**
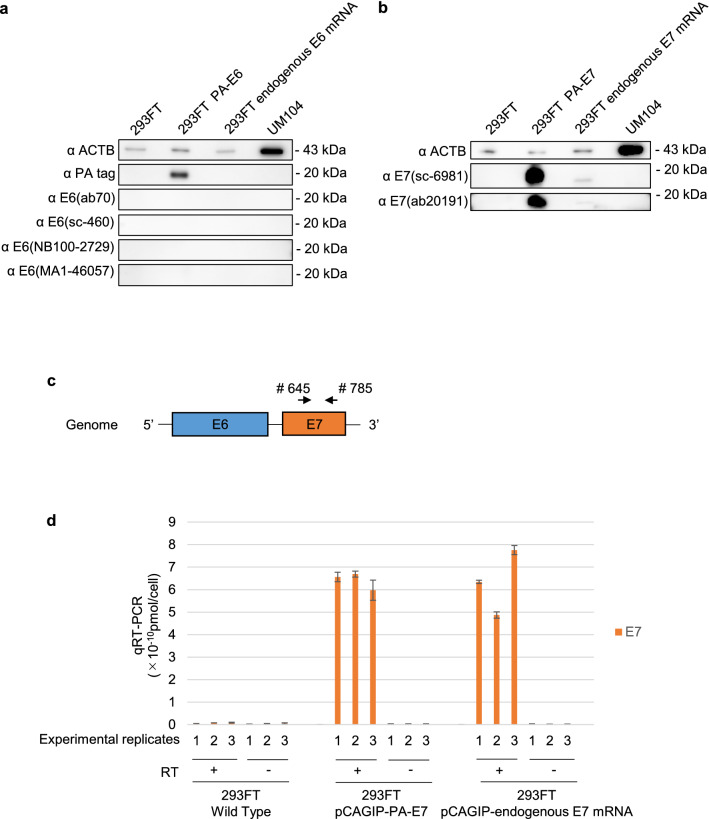
E7 protein expression in 293FT cells by overexpression of E7 mRNA having the endogenous structure. (**a**,**b**) Western blotting of E6 (**a**) and E7 protein (**b**) in 293FT cells (wild-type), 293FT cells transfected with pCAGIP-PA-E6/-PA-E7 or pCAGIP-endogenous E6/E7 mRNA, and UM104 cells. (**c**) E7 primers (# 645 and # 785) designed for binding within the E7 gene for use in qRT-PCR. (**d**) qRT-PCR analysis of E7 expression in 293 FT cells transfected with pCAGIP-PA-E7 or -endogenous E7 mRNA plasmids. The values for E7 expression per cell are shown. RT+, Reactions performed in the presence of reverse transcriptase. RT−, reactions performed in the absence of reverse transcriptase. The numbers on the x-axis show the experimental replicates.

