# Supplementary material for: Evaluation of HPV16 E7 expression in head and neck carcinoma cell lines and clinical specimens
Source: Sci Rep. 2020 Dec 17;10:22138. doi: 10.1038/s41598-020-78345-8 (PMC7747560; doi:10.1038/s41598-020-78345-8)
Supplement: Supplementary file 1 — Supplementary Information [file 41598_2020_78345_MOESM1_ESM.pdf]

Evaluation of HPV16 E7 expression in head and neck carcinoma cell lines and clinical specimens

Authors list

Koji Kitamura, Keisuke Nimura, Rie Ito, Kotaro Saga, Hidenori Inohara & Yasufumi Kaneda

Correspondence and requests for materials should be addressed to K. N.
Tel.: +81-6-6879-3901; Fax: +81-6-6879-3909; nimura@gts.med.osaka-u.ac.jp

Supplemental Information includes
Supplemental Figures 1-6

【Supplemental Figure 1】

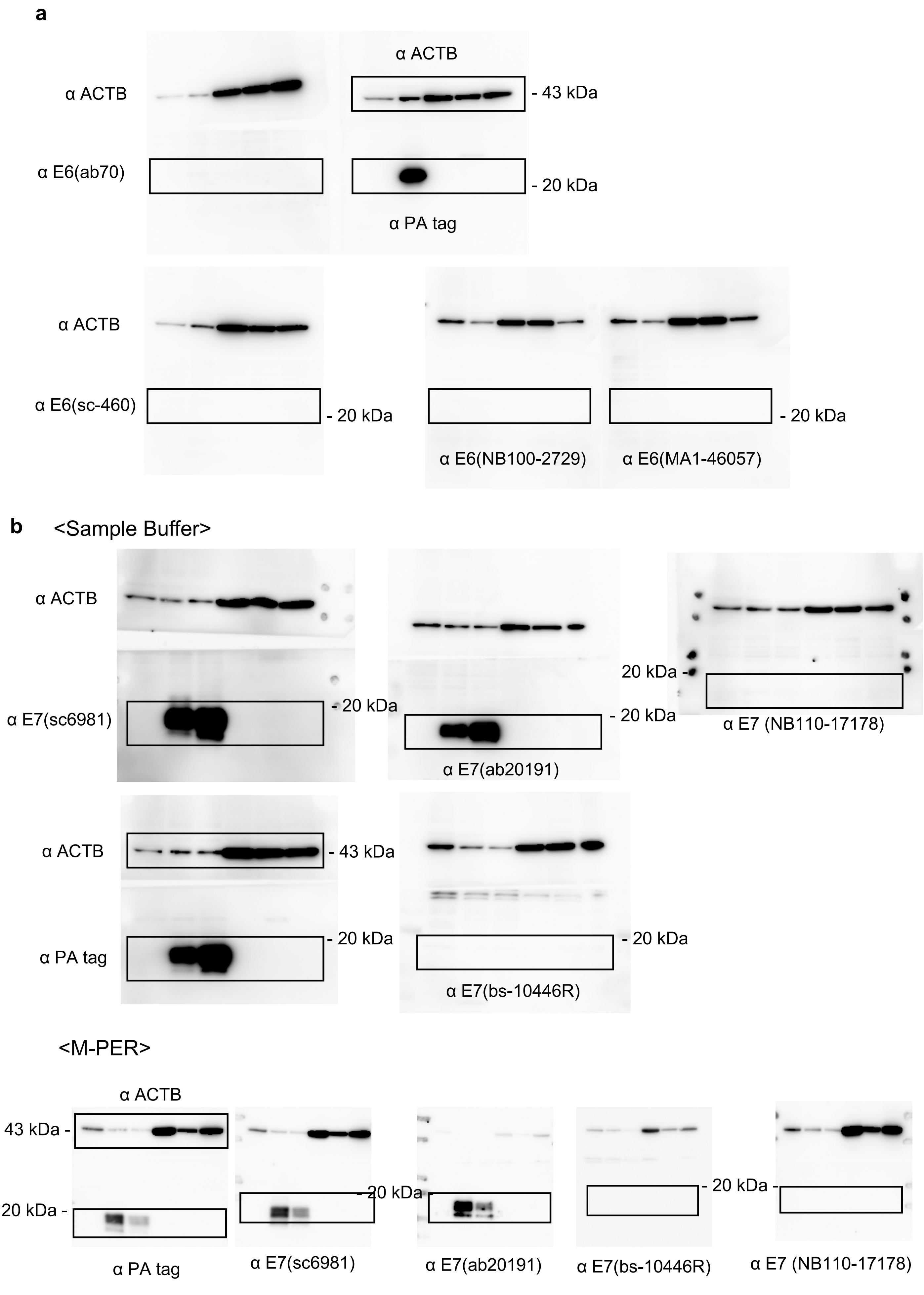

【Supplemental Figure 2】

a

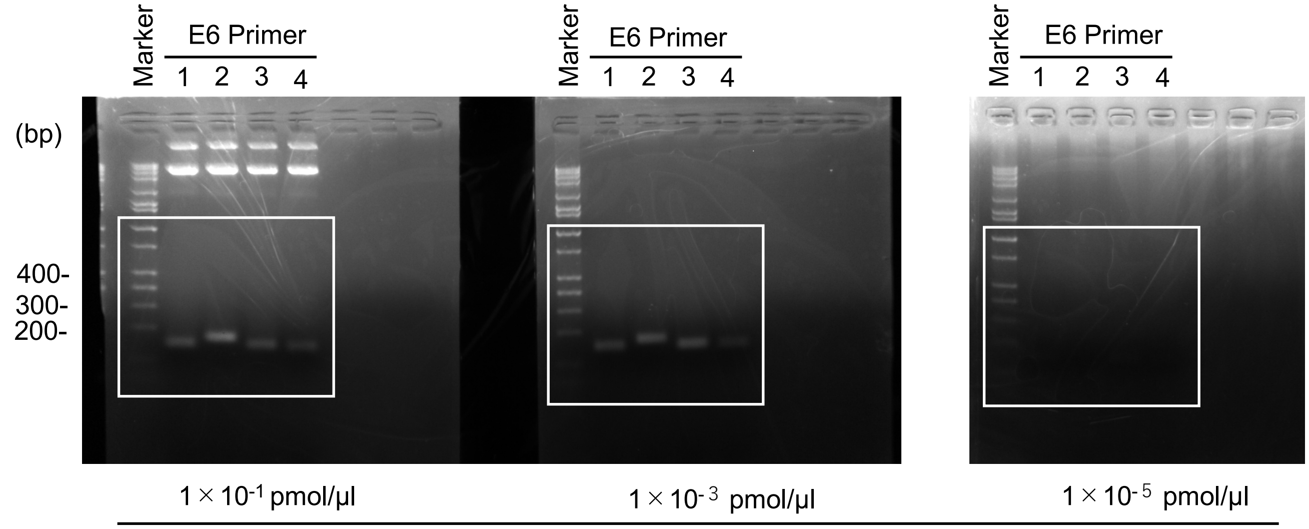

Template concentration (pmol/µl)

b

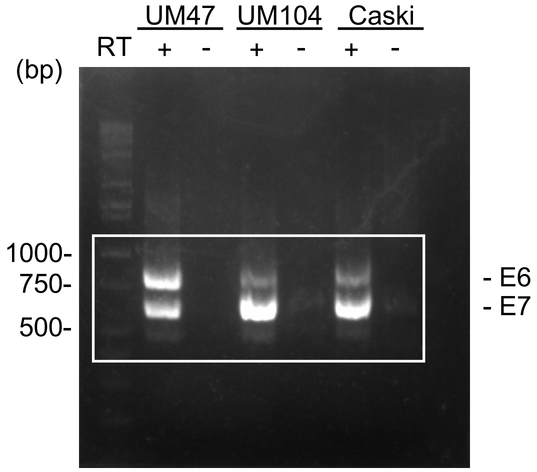

【Supplemental Figure 3】

a

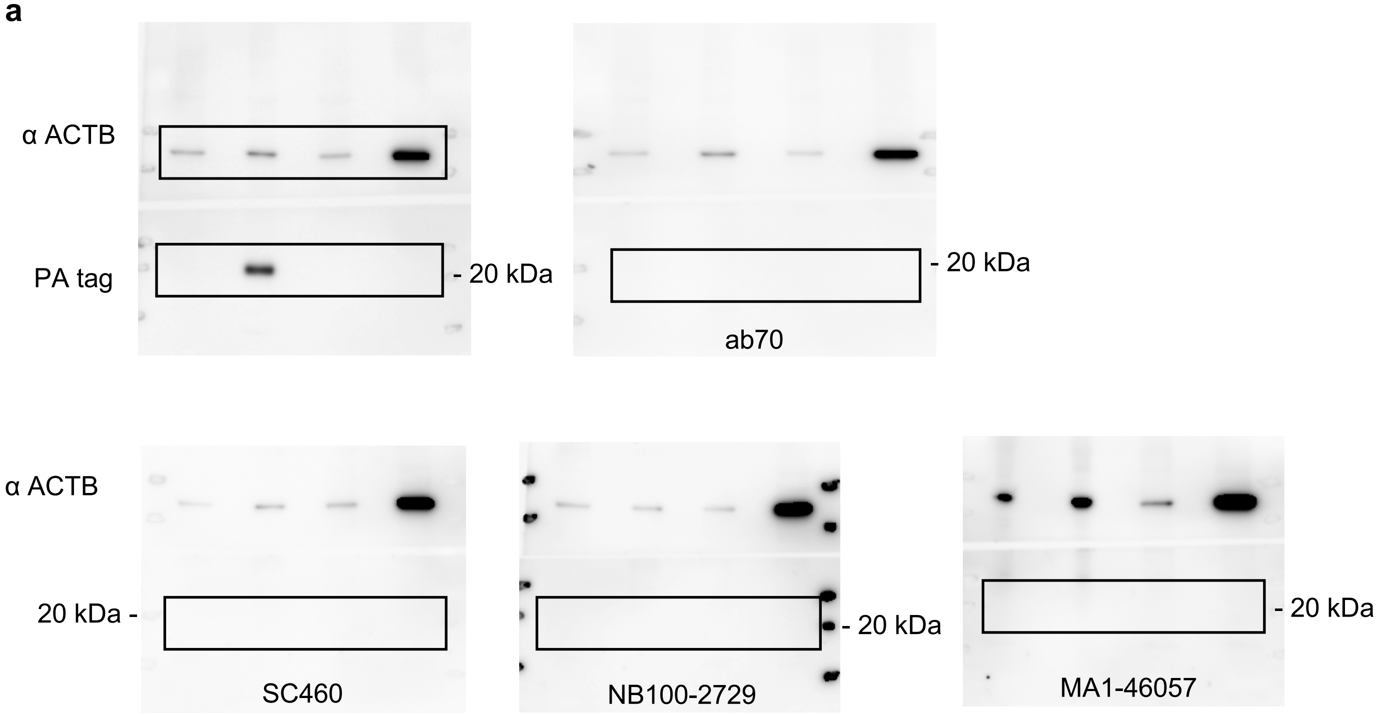

b

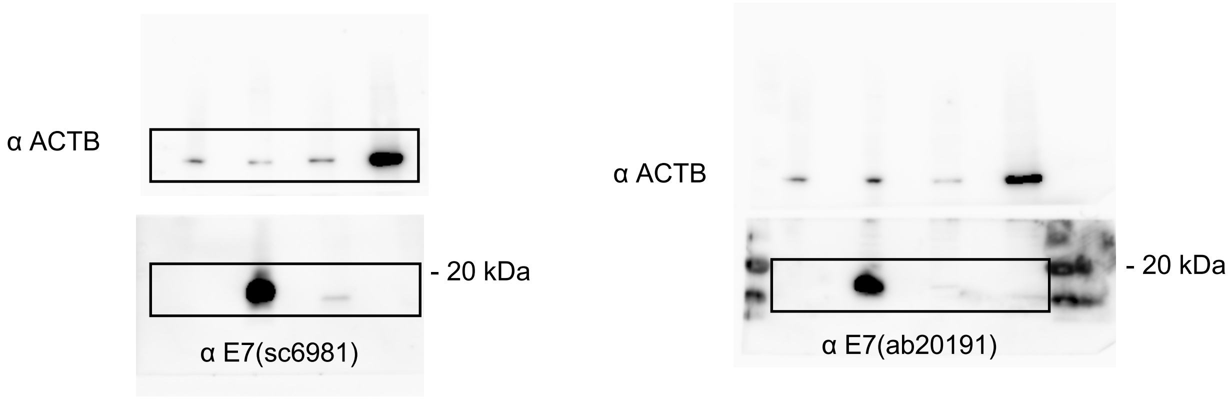

【Supplemental Figure 4】

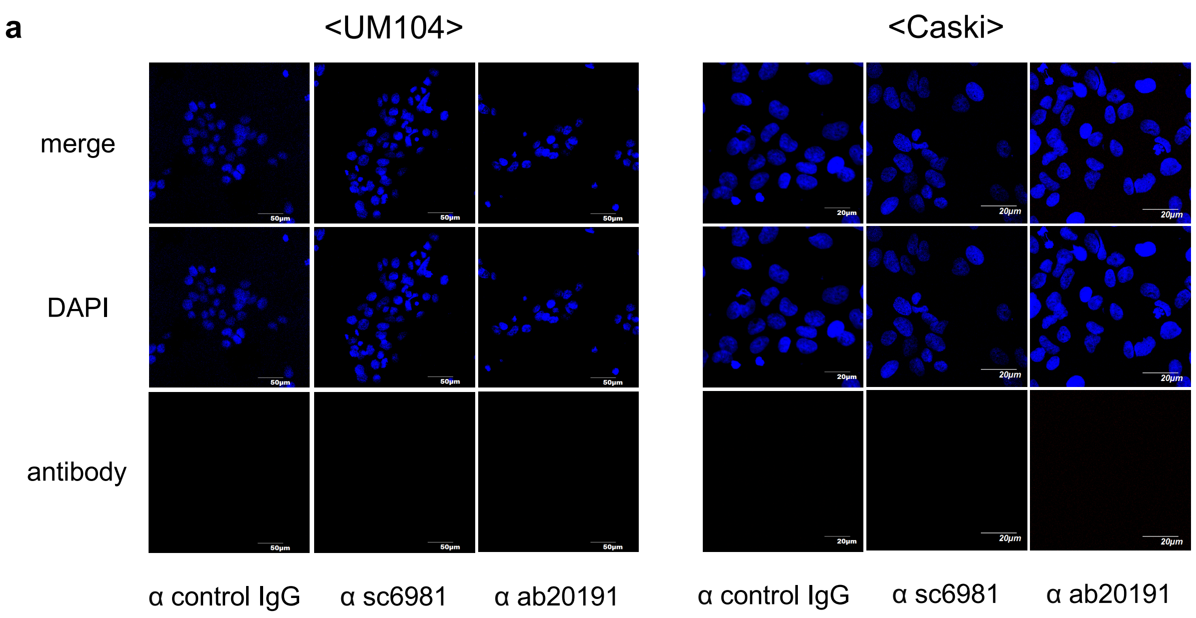

【Supplemental Figure 5】

a

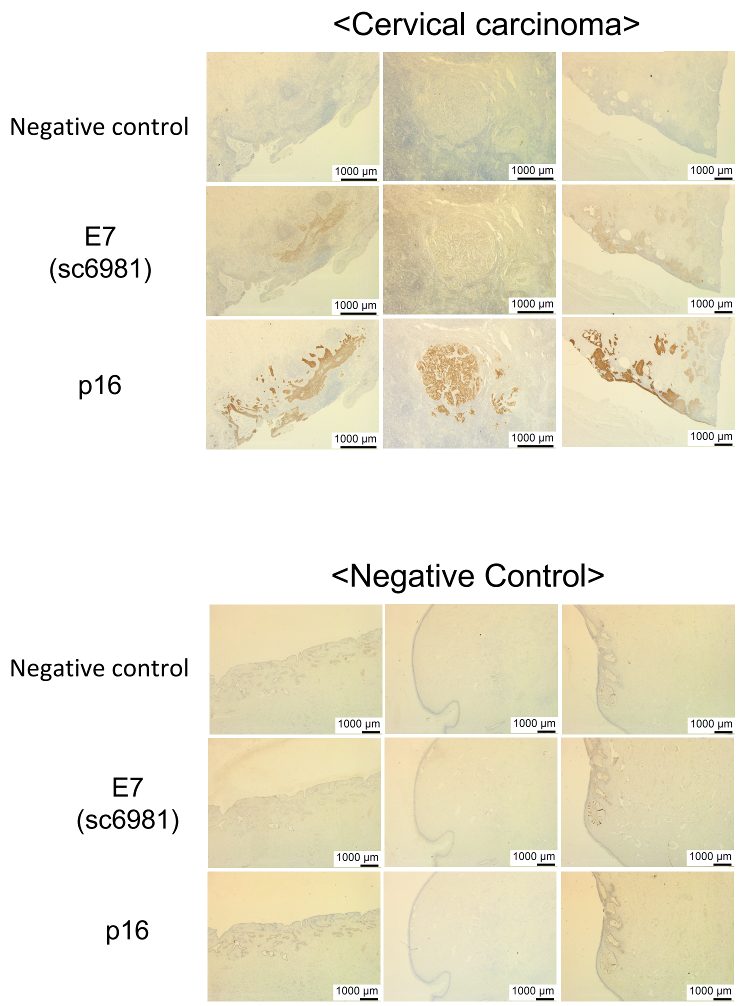

【Supplemental Figure 6】

a

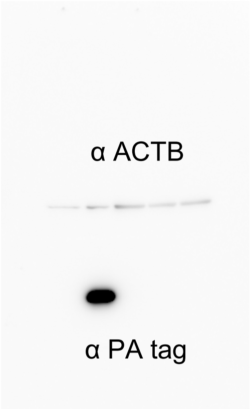

b

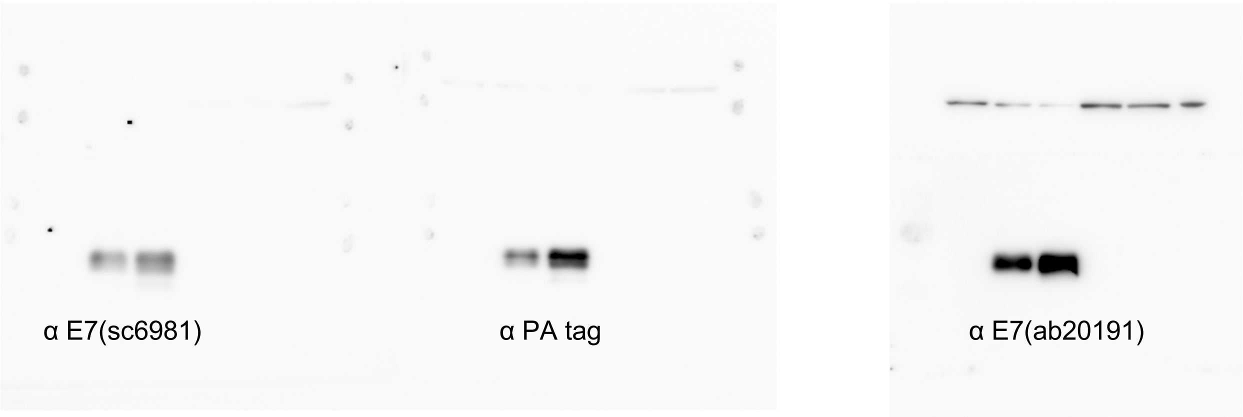

c

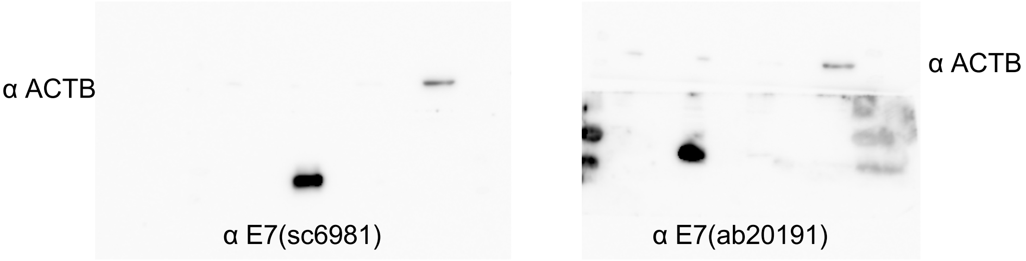

Supplemental Figure legends

Supplemental Figure 1. Raw data related to Figure 1. (**a**) raw data for Fig. 1b. (**b**) raw data for Fig. 1c. The enclosures of the black frame in Supplemental Fig. 1a and 1b were respectively cut off and re-assembled in Fig. 1b and 1c.

Supplemental Figure 2. Raw data related to Figure 2. (**a**) raw data for Fig. 2e. (**b**) raw data for Fig. 2g. The enclosures of the white frame in Supplemental Fig. 2a and 2b were respectively cut off and re-assembled in Fig. 2e and 2g.

Supplemental Figure 3. Raw data related to Figure 6. (**a**) raw data for Fig. 6a. (**b**) raw data for Fig. 6b. The enclosures of the black frame in Supplemental Fig. 3a and 3b were respectively cut off and re-assembled in Fig. 6a and 6b.

Supplemental Figure 4. Immunofluorescence analysis of E7 protein using HPV-positive cell lines, UM104 and Caski. (**a**) Anti-E7 antibodies (**a**, sc-6981 and **b,** ab20191) were used for immunofluorescence analysis of UM104 and Caski. DAPI was used for nuclear staining. Scale bar = 50 μm in UM104 and 20 μm in Caski.

Supplemental Figure 5. Immunohistochemical analysis of E7 protein in clinical samples of cervical carcinoma and normal cervical tissue. (**a**) Formalin-fixed and paraffin-embedded samples were stained using the indicated antibodies. Scale bar = 1 mm.

Supplemental Figure 6. Different exposure images related to Figure 1 and 6. (**a**) Different exposure images for Fig. 1b and Supplemental Fig. 1a. (**b**) different exposure images for Fig. 1c and Supplemental Fig. 1b. (**c**) different exposure images for Fig. 6b and Supplemental Fig. 3b.
